# Simultaneous Optimization of Microwave-Assisted Extraction of Phenolic Compounds and Antioxidant Activity of Avocado (*Persea americana* Mill.) Seeds Using Response Surface Methodology

**DOI:** 10.1155/2020/7541927

**Published:** 2020-08-17

**Authors:** Alexander Weremfo, Felix Adulley, Martin Adarkwah-Yiadom

**Affiliations:** ^1^Department of Biochemistry, School of Biological Sciences, University of Cape Coast, Cape Coast, Ghana; ^2^Drugs, Cosmetics and Forensic Department, Ghana Standards Board, Accra, Ghana

## Abstract

This study was designed to optimize three microwave-assisted extraction (MAE) parameters (ethanol concentration, microwave power, and extraction time) of total phenolics, total flavonoids, and antioxidant activity of avocado seeds using response surface methodology (RSM). The predicted quadratic models were highly significant (*p* < 0.001) for the responses studied. The extraction of total phenolic content (TPC), total flavonoid content (TFC), and antioxidant activity was significantly (*p* < 0.05) influenced by both microwave power and extraction time. The optimal conditions for simultaneous extraction of phenolic compounds and antioxidant activity were ethanol concentration of 58.3% (v/v), microwave power of 400 W, and extraction time of 4.8 min. Under these conditions, the experimental results agreed with the predicted values. MAE revealed clear advantages over the conventional solvent extraction (CSE) in terms of high extraction efficiency and antioxidant activity within the shortest extraction time. Furthermore, high-performance liquid chromatography (HPLC) analysis of optimized extract revealed the presence of 10 phenolic compounds, with rutin, catechin, and syringic acid being the dominant compounds. Consequently, this optimized MAE method has demonstrated a potential application for efficient extraction of polyphenolic antioxidants from avocado seeds in the nutraceutical industries.

## 1. Introduction

Avocado (*Persea americana* Mill.) belongs to the family of Lauraceae and is an important fruit crop endemic to the tropical and subtropical regions but presently cultivated worldwide. The food industry has shown remarkable interest in processing and enhancing the value of this crop due to its high economic importance. In addition to its pleasant sensory properties, there has been growing interest in the consumption of avocado-derived products owing to its high nutritional value and reported health-promoting and/or disease-preventing properties [1, 2]. The seed is a major by-product of avocado industry and is usually discarded with no further application [3]. In addition, this important by-product represents an environmental and waste management problem. The avocado seed constitutes up to 16% of the weight of the fruit [4] and is a rich source of polyphenols with antioxidant and antimicrobial properties [4–7]. Recent studies have demonstrated the antioxidant, anticancer, antidiabetic, anti-inflammatory, blood pressure reducing, antimicrobial, insecticidal, and dermatological activities of seed preparations [4, 8]. Due to their beneficial effects, avocado seeds can be an alternative inexpensive source of bioactive compounds, and an efficient extraction of important phenolics from the avocado waste could improve the economics of the avocado industry and minimize environmental impact.

The extraction of phenolic compounds from avocado seeds has been investigated in the last decades focusing mainly on conventional extraction methods such as maceration, Soxhlet, and heat reflux extraction methods. However, these methods are very time-consuming and require large quantities of solvents [9, 10]. Recently, several efficient and advanced extraction techniques including accelerated solvent extraction [6], ultrasound-assisted extraction [11], and supercritical fluid extraction [12] have been developed for the extraction of phenolic compounds from avocado seeds. Microwave-assisted extraction (MAE) is a green and effective extraction technique that uses microwave energy to heat polar solvent in contact with samples, by ionic conduction and dipole rotation, which improves cell wall destruction and increases solubility of compounds such as flavonoids [13–15]. MAE has gain popularity in recent times due to it benefits of improved efficiency, reduced extraction time, low solvent consumption, higher extraction rate, and high potential for automation [16, 17]. MAE technique has been used for the extraction of bioactive compounds from a wide variety of matrices, such as grapes [18], tomatoes [19], apple [20], and coffee [21]. However, the extraction of phenolic bioactive compounds from avocado seeds has not been evaluated using MAE.

The efficiency of the MAE process is usually affected by several variables such as extraction power, time, solvent composition, and solvent-to-sample ratio [18, 22–24]. It is therefore important to optimize these process variables to achieve maximum yield of bioactive compounds from the raw materials. In this study, a response surface methodology (RSM) was used to determine the effect of MAE process variables and their interactions to ensure maximal extraction efficiency. This method allows the optimization of all variables simultaneously and predicts the most efficient conditions with the use of a minimal number of experiments [25]. RSM has recently been used to optimize the extraction conditions of phenolics from various plants [26–28]. Thus, the objective of this study was to optimize MAE conditions to obtain maximum yield of phenolic antioxidants from avocado seeds. RSM was used to predict the effects of microwave power, extraction time, and ethanol concentration on total phenolic content (TPC), total flavonoid content (TFC), and antioxidant activity of avocado seed extract.

## 2. Materials and Methods

### 2.1. Chemicals

Folin–Ciocalteu phenol reagent; aluminium chloride (AlCl_3_·6H_2_O); sodium carbonate (Na_2_CO_3_); 2,2-diphenyl-1-picrylhydrazyl radical (DPPH); 2,2′-azinobis-(3-ethylbenzothiazoline-6-sulfonic acid) (ABTS); sodium nitrite (NaNO_2_); sodium hydroxide (NaOH); ethanol; and phenolic compounds (gallic acid, catechin, rutin, quercetin, 4-hydroxybenzoic acid, syringic acid, ferulic acid, vanillic acid, caffeic acid, and *p*-coumaric acid) were purchased from Sigma-Aldrich Corp. (St. Louis, MO, USA). All chemicals and solvents were of analytical grade.

### 2.2. Sample Preparation

Avocado fruits (*Persea americana* Mill. var. Hass), with adequate ripeness for consumption, were obtained from a local market at Bonyere (Ghana) in February 2019. The seeds were manually removed from the fruits, cleansed, sliced into small and thin size, and sun-dried for 12 days until no more weight loss was observed. The dried seeds were milled into fine powder using a blender, and the particle size was standardized using a 250 *μ*m sieve. The moisture content of the dried avocado seeds was 8.9%. The powdered sample was stored at −20°C in airtight bags until being used.

### 2.3. Experimental Design

A face-centred central composite design was used to optimize three independent microwave parameters: ethanol concentration (%, *X*_1_), microwave power (W, *X*_2_), and extraction time (min, *X*_3_) of four dependent variables: total phenolic content (*Y*_TPC_), total flavonoid content (*Y*_TFC_), DPPH scavenging activity (*Y*_DPPH_), and ABTS scavenging activity (*Y*_ABTS_). These independent microwave parameters were selected due to their significant influence on the efficiency of MAE [18, 22–24]. Generally, ethanol and methanol are better solvents for extraction of phenolic compounds. Considering the potential use of this product in food industry, ethanol was selected as the solvent in this study. The independent variables were coded at three levels, and their actual values selected based on literature data and preliminary experimental results. The independent variables and their related codes and levels are displayed in Table 1. A total of 17 experimental runs were performed randomly, which included three replicates at the centre point (Table 2), and all the experiments were replicated thrice to improve the analysis. Regression analysis for the experiment data was performed and was fitted into a second-order polynomial model:

(1) $$\begin{array}{c} Y=\beta_{o}+\overset{k}{\underset{i=1}{\sum }}\beta_{i}x_{i}+\overset{k=1}{\underset{i=1,i<j}{\sum }}\overset{k}{\underset{j=2}{\sum }}\beta_{ij}x_{j}+\overset{k}{\underset{i=2}{\sum }}\beta_{ii}x_{ii}^{2}, \end{array}$$

where *β*_0_, *β*_*i*_, *β*_*ii*_, and *β*_*ij*_ are the regression coefficients; *x*_*i*_ and *x*_*j*_ are the coded levels of independent variables affecting the dependent response *Y*; and *k* is the number of parameters.

### 2.4. Extraction Process

#### 2.4.1. Microwave-Assisted Extraction (MAE)

MAE was performed using a domestic microwave oven system (Kenwood K30CSS14 Microwave, China) operating at 800 W maximum power and a frequency of 2450 MHz. The apparatus was equipped with a digital control system for irradiation time and microwave power. The oven was modified in order to condense the vapor generated during extraction into the sample. 1 g of avocado seeds powder was stirred in 20 mL aqueous ethanol, and the mixture was irradiated using the microwave system. The MAE extraction parameters were microwave power (80–400 W), extraction time (1–5 min), and ethanol concentration (40–80%). Thereafter, the sample was filtered using a vacuum pump, and the liquid extract was collected and stored at 4°C until further use.

#### 2.4.2. Conventional Solvent Extraction (CSE)

Phenolic compounds in avocado seeds were extracted using a CSE method optimized by Gómez et al. [29]. Briefly, 1 g of avocado seeds powder was mixed with 60 mL of 56% ethanol (v/v), and the mixture was kept in a thermostatic water bath (Grant W14, Cambridge, England) at 63°C, with shaking for 23 min. After cooling, the mixture was centrifuged at 2500 rpm for 10 min, and the supernatant was recovered through filtration and stored at 4°C until further use.

### 2.5. Phytochemical Analysis

#### 2.5.1. Determination of Total Phenolic Content (TPC)

TPC of the avocado seed extract was determined using the Folin–Ciocalteu method [16]. The extract (100 *μ*L) was mixed with 750 *μ*L of a 10-fold diluted Folin–Ciocalteu reagent followed by 750 *μ*L of sodium carbonate (7.5%, w/v). The mixture was incubated in dark at room temperature (27°C) for 90 min, and its absorbance measured at 725 nm using an UV-Vis spectrophotometer (Labomed Spectro UVD 3200, USA) against the blank. Gallic acid was used for the calibration curve (Figure S1). The results were expressed as mg of gallic acid equivalent (GAE) per gram of dry weight (dw) of avocado seeds.

#### 2.5.2. Total Flavonoid Content (TFC)

The flavonoid content in the extract was determined by the aluminium chloride method [30]. Briefly, 0.5 mL of extract was diluted with 1.5 mL of distilled water, 0.5 mL of 10% (w/v) aluminium chloride, and 0.1 mL of potassium acetate (1 M). The final volume was made up to 5 mL with distilled water, and the mixture kept at room temperature for 30 min. The absorbance was measured at a wavelength of 415 nm against blank (AlCl_3_ solution) after 30 min of equilibrium. The TFC was quantified using quercetin standard curve (Figure S2) and estimated as mg of quercetin equivalent (QE) per gram of dry weight (dw) of avocado seeds.

### 2.6. Antioxidant Activity

#### 2.6.1. DPPH Radical Scavenging Activity

The DPPH assay was performed as described by Pandey et al. [30]. The extract (1 mL) was mixed with 3 mL of DPPH solution (4 mL of stock DPPH solution in 96 mL of 80% methanol), and the mixture was kept in dark for 30 min at room temperature. The absorbance of the mixture was measured at 520 nm using UV-Vis spectrophotometer (Labomed Spectro UVD 3200, USA). A mixed solution of 1 mL ethanol and 3 mL DPPH solution was used as the blank. Antioxidant activity of the extract was expressed as the percent inhibition, according to the following equation:

(2) $$\begin{array}{c} \%\text{inhibition}=\;\frac{A_{\text{control}}-\;A_{\text{sample}}}{A_{\text{control}}}\times 100, \end{array}$$

where *A*_control_ is the absorbance value of the blank and *A*_sample_ is the absorbance of extract and DPPH solution.

#### 2.6.2. ABTS Radical Scavenging Activity

ABTS radical scavenging ability of the avocado seed extract was evaluated using a spectrophotometric method as described by Dahmoune et al. [16]. A radical solution (7 mM ABTS and 2.45 mM potassium persulfate in equal proportions) was prepared and left to stand in the dark at room temperature (27°C) for 16 h until the reaction was completed, and the absorbance was stable at 734 nm. This solution was diluted with ethanol (80%) till an absorbance value of 0.70 ± 0.02 at 734 nm was obtained. The extract (0.1 mL) was mixed with 3.9 mL of diluted ABTS solution and kept in dark for 15 min at room temperature. The absorbance was measured at 734 nm against blank (diluted ABTS solution) using UV-Vis spectrophotometer (Labomed Spectro UVD 3200, USA). The antioxidant activity of the extract was expressed as percent inhibition, according to

(3) $$\begin{array}{c} \%\text{inhibition}=\;\frac{A_{\text{control}}-\;A_{\text{sample}}}{A_{\text{control}}}\times 100, \end{array}$$

where *A*_control_ is the absorbance value of the blank and *A*_sample_ is the absorbance of extract and ABTS solution.

### 2.7. HPLC Analysis

Phenolic compounds present in the optimized extract were analyzed using Shimadzu UFLC chromatographic system (Shimadzu Corporation, Kyoto, Japan), equipped with two LC-20AD pumps and SPD-20AV ultraviolet-visible detector. The separation of the compounds was performed using Luna C18 column (150 mm × 4.6 mm, 3 *μ*m) at a column temperature of 30°C. The mobile phase consisted of A (1% acetic acid in acetonitrile) and B (1% acetic acid in water) with gradient elution 0–3 min (9% A), 3–37 min (9–68% A), 37–39 min (68% A), and 39–40 min (69–9% A). The flow rate was 0.8 mL/min, and the injection volume was 5 *μ*L. Each standard solution and sample was analyzed in triplicate. The peaks were detected by UV at wavelength of 280 nm according to the scanning mode of the UV detector. The phenolic compounds were identified by comparing their retention times with corresponding standards. All the identified compounds were quantified by external standard method using calibration curves, and their concentrations were expressed as mg/100 g·dw.

### 2.8. Statistical Analysis

Statistical analysis and response surface plots were performed using the Design-Expert software (version 11.0, Stat-Ease, Inc., MN, USA). Data were analyzed using analysis of variance (ANOVA) at 95% confidence level.

## 3. Results and Discussion

### 3.1. Model Fitting

A central composite design (CCD) was used to study the effects and interactions of MAE parameters (ethanol concentration, microwave power, and extraction time) on TPC, TFC, DPPH, and ABTS. The experimental design matrix with corresponding responses is presented in Table 1. The experimental values obtained ranged from 47.25 to 89.39 mg·GAE/g for TPC, 0.66 to 21.45 mg·QE/g for TFC, 22.93 to 79.76% DPPH inhibition, and 11.21 to 80.32% ABTS inhibition (Table 2). The values showed considerable dependence on the extraction conditions, which suggests the need to optimize the extraction process. Quadratic polynomial models were developed, and the adequacy and fitness of the models were evaluated by ANOVA. The ANOVA results revealed that the four models were highly significant (*P* < 0.0001) for TPC, TFC, DPPH, and ABTS (Table 3). The respective values of *R*^2^, Adj-*R*^2^ and Pred-*R*^2^ for TPC (0.9758, 0.9446, and 0.8086, respectively), TFC (0.9875, 0.9715, and 0.8679, respectively), DPPH (0.9912, 0.9800, and 0.9372, respectively), and ABTS (0.9899, 0.9770, and 0.9255, respectively) were all close to 1, indicating good correlation between the predicted and the actual results [31]. Moreover, the low values of coefficient of variation (CV, %: 3.55, 9.28, 4.83, and 5.98) suggested that the experimental values were reliable and reproducible [32, 33]. Furthermore, the lack of fit values were not significant (*P* > 0.05), indicating the adequacy of the model in predicting MAE of phenolic compounds and antioxidant activity of avocado seeds.

### 3.2. Influence of the Extraction Parameters on Total Phenolic Content

The TPC in avocado seed extract varied from 47.25 to 89.39 mg·GAE/g (Table 2). The lowest yield was achieved at ethanol concentration of 80% and microwave power of 80 W after 1 min of extraction, while the highest yield was obtained at ethanol concentration of 60% and microwave power of 400 W after 3 min of extraction time. Table 3 shows that microwave power (*X*_2_) and extraction time (*X*_3_) had significant (*P* < 0.05) positive effect on TPC and the most significant factor is microwave power. The quadratic effect (*X*_1_^2^, *X*_2_^2^ and *X*_3_^2^) also had significant (*P* < 0.05) influence on TPC under MAE. There was a significant (*P* < 0.05) interaction between ethanol concentration and extraction time (*X*_1_*X*_3_), as well as microwave power and extraction time (*X*_2_*X*_3_). The second-order polynomial equation for TPC was expressed as

(4) $$\begin{array}{c} Y_{\text{TPC}}=83.19-1.20X_{1}+8.17X_{2}+5.31X_{3}-0.38X_{1}X_{2}+2.19X_{1}X_{3}-2.17X_{2}X_{3}-5.55X_{1}^{2}-4.37X_{2}^{2}-7.46X_{3}^{2}. \end{array}$$

**Figure 1:**
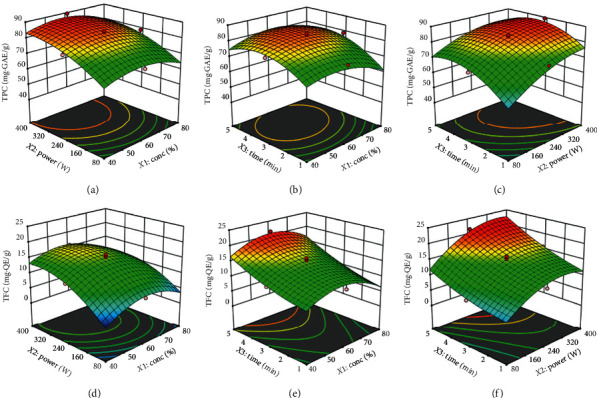
Response surface plot showing the interactive effect of MAE variables on TPC ((a)–(c)) and on TFC ((d)–(f)).

The effects of the independent variables and their mutual interactions on TPC can be seen on the three-dimensional response surface curves shown in Figures 1(a)–1(c). MAE of TPC from avocado seeds increased initially and decreased as the ethanol concentration increased (Figures 1(a) and 1(b)). Similar observation was reported for MAE of polyphenols from *Coriolus versicolor* mushroom [22], from chokeberries [23], from *Myrtus communis* L. leaves [16] and from blueberry leaves [34]. This significant (*p* < 0.01) quadratic effect of ethanol concentration on TPC (Table 3) could be explained by the heightened degree of sample cell membrane breakage and improved phenolic compounds solubility by the initial increase in ethanol concentrations [35, 36]. However, as ethanol concentration continues to increase, the polarity of the solvent changes, which may lead to increased impurities being extracted [35], therefore reducing the amount of total phenolic compounds extracted. Also increased diffusion resistance due to coagulation of proteins at high ethanol concentrations may prevent dissolution of polyphenols and influences the extraction rate [36].

As shown in Figure 1(a), microwave power had significant influence on TPC than ethanol concentration and this may be attributed to the increased solubility of phenolic compounds as a result of increasing power which promotes cell rupture and enhances exudation of phenolic compounds into the extracting solvent [37]. Ozbek et al. [38] reported similar behaviour for MAE of TPC from pistachio hull. The extraction time was an important parameter that influenced the extraction of TPC. As shown in Figures 1(a) and 1(b)), the extraction of TPC increased with increasing extraction time to about 4 min, beyond which a decrease in TPC was observed. This result is in agreement with that reported from Calop pulp [39]. Extended extraction time was expected to favour the extraction of phenolic compounds, since enough time is required for solvent penetration into the plant tissue, dissolving the compounds and subsequently diffusing out to the extraction medium [40]. However, at longer extraction time, the extracted yields decreased due to increased dissolution of polymer matrix, which causes an increase in viscosity and thereby encapsulating the extracted compounds [41]. In addition, long extraction time may increase exposure to light and oxygen which will eventually result in the oxidation of phenolic compounds [42]. According to ANOVA analysis (Table 3), the interactive effect of ethanol concentration and extraction time (*X*_1_*X*_3_) had significant positive influence (*p* < 0.05) on TPC. As shown in Figure 1(b), the extraction of TPC increased with increasing ethanol concentration and extraction time to about 60% and 4 min, respectively, after which increasing ethanol concentration and extraction time caused a decrease in the recovery of TPC.  Figure 1(c) illustrates the effect of microwave power and extraction time on TPC. This significant (*p* < 0.05) positive interaction (Table 2) is in agreement with earlier reports [43, 44]. Increasing microwave power increased TPC as extraction time increases (1–3 min). This phenomenon could be explained by the enhanced mass transfer rate and solubility of phenolic compounds due to decreasing surface tension and solvent viscosity with increasing microwave power, which improve sample wetting and matrix penetration, respectively, thereby enhancing extraction efficiency [16, 45, 46]. However, at high levels of microwave power (320–400 W), increasing the extraction time after 4 min decreased TPC which may be due to degradation of certain phenolic compounds [16].

### 3.3. Influence of the Extraction Parameters on Total Flavonoid Content

The predictive equation for the relationship between TFC and the extraction parameter was expressed as follows:

(5) $$\begin{array}{c} Y_{\text{TFC}}=15.04-0.40X_{1}+4.40X_{2}+4.60X_{3}-1.16X_{1}X_{2}+0.48X_{1}X_{3}+1.06X_{2}X_{3}-4.32X_{1}^{2}-3.56X_{2}^{2}+1.17X_{3}^{2}. \end{array}$$

As shown in Table 3, microwave power and extraction time exhibited a highly significant (*p* < 0.001) positive linear effect, while the quadratic terms of ethanol concentration and microwave power showed significant (*p* < 0.01) negative effect on the extraction of TFC from avocado seeds. The same linear and quadratic effects were observed for TPC extraction, which suggests that similar factors affected the extraction of TFC from avocado seeds. This is expected as flavonoids represent a subgroup of polyphenols. The interaction of microwave power and extraction time (*X*_2_*X*_3_) had a significant (*p* < 0.05) positive effect on TFC. At lower microwave powers, increasing extraction powers gradually increased TFC value over time (Figure 1(f)). This significant (*p* < 0.05) interaction of microwave power and extraction time (*X*_2_*X*_3_) is tentatively explained by the low rate of mass transfer at low microwave powers, which would require more time for the phenolic compounds to dissolve from the avocado seeds into the solution. At higher microwave powers, the dissolution of phenolic compounds can reach equilibrium in a relatively short time, hence the extraction of TFC are not readily affected by changes in the extraction time. Ethanol concentration was the least important factor as it did not show a significant effect on TFC (Table 3). However, the significant (*p* < 0.05) negative interaction of ethanol concentration and microwave power (*X*_1_*X*_2_) on the extraction of TFC suggested that optimal microwave power values increase as ethanol concentration decreases (Figure 1(d)).

### 3.4. Influence of the Extraction Parameters on Antioxidant Activity

The antioxidant activity of the avocado seed extract was determined using ABTS and DPPH assays. The results in Table 3 show that the ABTS scavenging activity was influenced by ethanol concentration, microwave power and extraction time, while DPPH activity depended on microwave power and extraction time. The model equation for antioxidant activity can be represented as follows:

(6) $$\begin{array}{c} Y_{\text{ABTS}}=67.44-2.27X_{1}+11.97X_{2}+11.43X_{3}+0.01X_{1}X_{2}+0.47X_{1}X_{3}-0.34X_{2}X_{3}-22.82X_{1}^{2}-0.80X_{2}^{2}-7.35X_{3}^{2},\\ Y_{\text{DPPH}}=53.93-1.61X_{1}+14.90X_{2}+12.13X_{3}-0.81X_{1}X_{2}-1.13X_{1}X_{3}+5.82X_{2}X_{3}-4.63X_{1}^{2}-5.93X_{2}^{2}+1.00X_{3}^{2}. \end{array}$$

**Figure 2:**
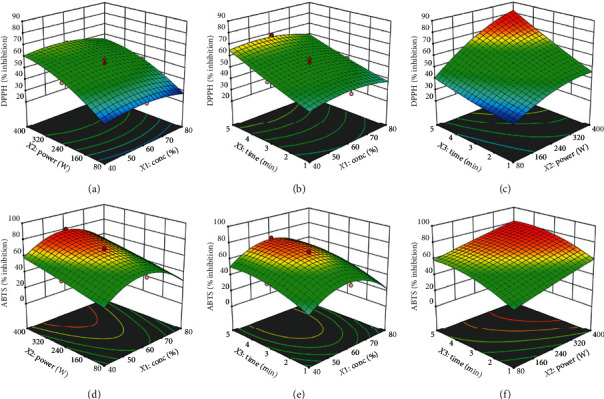
Response surface plot showing the interactive effect of MAE variables on DPPH and ABTS activity.

The linear effects of microwave power and extraction time showed a highly significant (*p* < 0.001) positive effect on ABTS scavenging activity, while ethanol concentration exhibited significant (*p* < 0.05) negative effect on ABTS. Moreover, the quadratic effects of ethanol concentration (*X*_1_^2^) and extraction time (*X*_3_^2^) showed highly significant (*p* < 0.001) and moderately significant (*p* < 0.01) negative effects on ABTS activity, respectively (Table 3). As shown in Figure 2(e), increasing ethanol concentration above 60% resulted in a quadratic decrease in ABTS activity. Interestingly, there was no significant interactive impact (*p* > 0.05) of *X*_1_*X*_2_, *X*_1_*X*_3_, or *X*_2_*X*_3_ on ABTS scavenging activity (Table 3). This indicates that the ABTS scavenging activity of the extract was individually affected by ethanol concentration, microwave power, and extraction time and not by their interaction.

In case of DPPH antioxidant activity, both microwave power and extraction time showed highly significant (*p* < 0.001) positive linear effect. The quadratic effects of the ethanol concentration (*X*_1_^2^) (*p* < 0.05) and microwave power (*X*_2_^2^) significantly (*p* < 0.01) influenced DPPH scavenging activity (Table 3). Moreover, increasing both microwave power and extraction time resulted in significant positive interactive effect on DPPH activity (Figure 2(c)). Thus, the longer the extraction time, the better the DPPH scavenging activity of the extract. Similar observation was reported by Garrido et al. [47] from chardonnay grape marc.

Although both ABTS and DPPH scavenging activities exhibited relatively similar patterns, the minor differences could be due to the present of various phenolic compounds in the extract, which exert different kinetics and reaction mechanisms to different antioxidant activity [30]. Similar findings have been reported from vine pruning residues [48] and from rhizomes of *Rheum moorcroftianum* [30].

### 3.5. Optimization of Extraction Conditions and Verification of Predictive Model

The optimal conditions for simultaneous extraction of maximum phenolic compounds (TPC and TFC) and antioxidant activity (DPPH and ABTS) from dry avocado seeds were predicted by maximizing the desirability of the responses using Design-Expert software trial version 11.0 (Stat-Ease, Inc.). The optimal microwave extraction conditions for optimum TPC, TFC, DPPH, and ABTS in a single experiment were determined to be as ethanol concentration of 58.3%, microwave power of 400 W, and extraction time of 4.8 min with desirability of 0.955. The numerical optimization provided the maximum predicted values of 83.90 mg·GAE/g for TPC, 21.84 mg·QE/g for TFC, 75.67% DPPH inhibition, and 82.66% ABTS inhibition. Experiments were performed under the optimized conditions, and the results are presented in Table 4. The experimental values agreed with the predicted values, confirming the reliability of the model obtained by CCD in predicting the contents of phenolic compounds and antioxidant activity using MAE.

### 3.6. Comparison of MAE with CSE

The results of TPC, TFC, and antioxidant activity from avocado seeds by MAE and CSE are shown in Table 5. The MAE method significantly (*p* < 0.05) enhanced the extraction of phenolic compounds and antioxidant activity as compared to CSE. In addition to improved extraction efficiency, solvent consumption and extraction time were significantly reduced by MAE in comparison with CSE. Using ultrasound-assisted extraction, a TPC value of 57.3 mg·GAE/g was obtained from avocado seeds [11]. The fast and efficient extraction of phenolic compounds from avocado seeds by MAE could be explained by the rapid heat generation by microwave energy which causes destruction of the cellular matrix and enhances the release of phenolic compounds [13, 14] and hence antioxidant activity.

### 3.7. HPLC Analysis of Phenolic Compounds in Avocado Seed Extract

**Figure 3:**
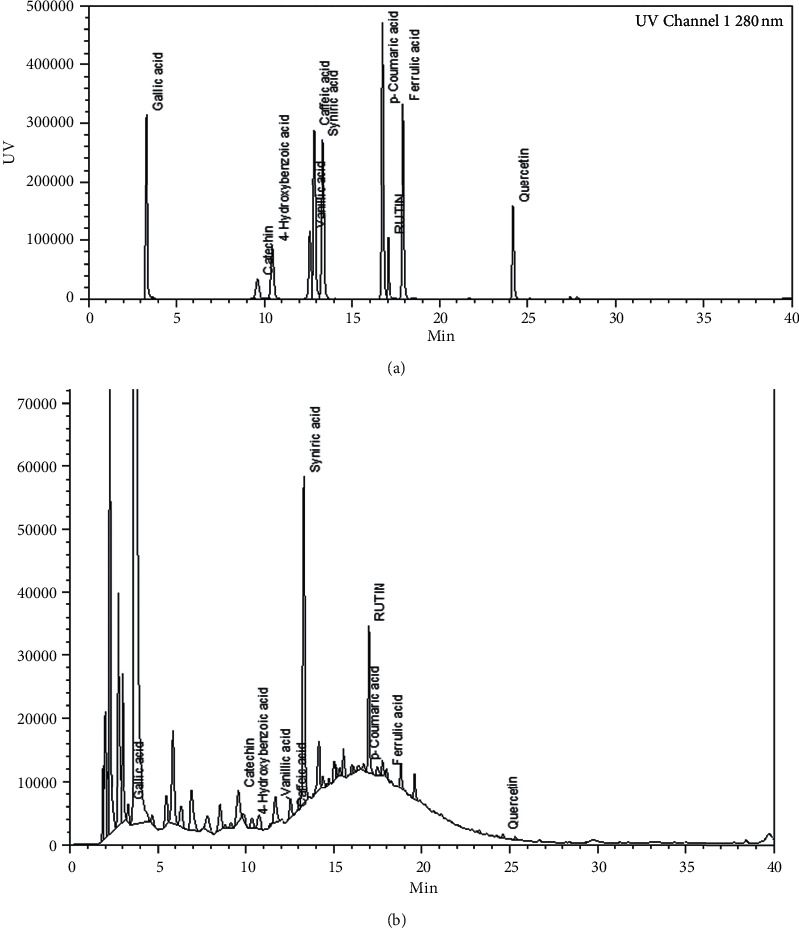
HPLC chromatograms of (a) mixed phenolic standards and (b) avocado seed extract recorded at 280 nm.

Ten phenolic compounds contained in the optimized extract of avocado seeds were identified by HPLC at wavelength of 280 nm (Figure 3). The identified compounds were gallic acid, catechin, 4-hydroxybenzoic acid, vanillic acid, caffeic acid, syringic acid, *p*-coumaric acid, rutin, ferulic acid, and quercetin. Most of these compounds have previously been identified in avocado seeds [3, 49, 50]. The content of each phenolic compound in this extract was quantified (Table 6). The most abundant compounds in this extract were rutin (71.67 mg/100 g), catechin (52.46 mg/100 g), and syringic acid (45.87 mg/100 g). The concentration of catechin, which is one of the most abundant phenolic compounds in avocado seeds, was higher than the previously reported value of 25.84 mg/100 g [50]. This may be due to, among other things, the extraction technique employed. Most of the identified phenolic compounds have shown significant free radical scavenging activity [30, 51]; hence the combined effects of these phenolic compounds may be partly responsible for the antioxidant activity observed in the extract obtained by MAE.

## 4. Conclusion

In this study, three parameters of MAE were successfully optimized for the maximum extraction of polyphenolic compounds and antioxidant activity of avocado seeds using RSM. The results indicate that both microwave power and extraction time significantly influenced the extraction of phenolic compounds (TPC and TFC) and antioxidant activity (DPPH and ABTS). The optimal conditions for simultaneous extraction of maximum phenolic compounds (TPC and TFC) and antioxidant activity (DPPH and ABTS) from avocado seeds were ethanol concentration of 58.3%, microwave power of 400 W, and extraction time of 4.8 min. Under these conditions, the experimental results agreed with the predicted values. MAE revealed clear advantages over CSE in terms of high extraction efficiency and antioxidant activity of extract within the shortest extraction time. Furthermore, ten phenolic compounds have been identified and quantified from this extract. The predominant phenolic compounds in the avocado seed extract include rutin, catechin, and syringic acid. Thus, this optimized MAE method could be beneficial for the extraction and analysis of polyphenolic antioxidants from avocado seeds for industrial purposes.

## Figures and Tables

**Table 1:** Three levels of the three variables of the extraction process.

Independent variables	Symbols	Coded levels		
		−1	0	1
Ethanol concentration (%)	*X*_1_	40	60	80
Microwave power (W)	*X*_2_	80	240	400
Extraction time (min)	*X*_3_	1	3	5

**Table 2:** Central composite design (CCD) with observed response of the dependent variables from MAE of avocado seeds.

Independent variables				Phenolic compounds		Antioxidant activity	
Run order	*X*_1_ (%)	*X*_2_ (W)	*X*_3_ (min)	TPC (mg·GAE/g)	TFC (mg·QE/g)	DPPH (%inhibition)	ABTS (%inhibition)
1	40	80	1	52.99	0.98	22.93	17.59
2	80	80	1	47.25	0.66	24.86	11.21
3	40	400	1	74.64	8.89	44.31	39.6
4	80	400	1	64.76	5.90	39.88	35.63
5	40	80	5	65.94	5.59	38.23	37.62
6	80	80	5	66.34	9.14	32.52	35.47
7	40	400	5	76.29	19.70	79.76	60.66
8	80	400	5	77.83	16.65	73.95	56.18
9	40	240	3	77.06	10.92	49.64	46.40
10	80	240	3	78.71	9.70	47.56	40.66
11	60	80	3	68.73	6.43	32.5	50.77
12	60	400	3	89.39	15.70	62.11	80.32
13	60	240	1	72.79	10.14	39.8	44.81
14	60	240	5	79.16	21.45	68.66	73.18
15	60	240	3	83.52	15.48	54.35	68.92
16	60	240	3	80.45	15.23	52.59	67.44
17	60	240	3	84.66	16.10	57.68	70.33

*X*_1_ = ethanol concentration; *X*_2_ = microwave power; *X*_3_ = extraction time; TPC = total phenolic content; TFC = total flavonoid content; DPPH = 2,2-diphenyl-1-picrylhydrazyl radical scavenging ability; ABTS = 2,2′-azinobis-(3-ethylbenzothiazoline-6-sulfonic acid) scavenging ability.

**Table 3:** Regression coefficient (*β*) and analysis of variance (ANOVA) of the predicted second-order polynomial models for phenolic compounds and antioxidant activity.

Factor	Coefficient (*β*)			
	TPC	TFC	DPPH	ABTS
Intercept	83.19	15.04	53.93	67.44
*Linear*				
*X*_1_-conc	−1.20	−0.40	−1.61	−2.27^*∗*^
*X*_2_-power	8.17^*∗∗∗*^	4.40^*∗∗∗*^	14.90^*∗∗∗*^	11.97^*∗∗∗*^
*X*_3_-time	5.31^*∗∗*^	4.60^*∗∗∗*^	12.13^*∗∗∗*^	11.43^*∗∗∗*^
*Interaction*				
*X*_1_ * X*_2_	−0.3750	−1.16^*∗*^	−0.8075	0.01
*X*_1_ * X*_3_	2.19^*∗*^	0.48	−1.13	0.47
*X*_2_ * X*_3_	−2.17^*∗*^	1.06^*∗*^	5.82^*∗∗*^	−0.34
*Quadratic*				
*X*_1_^2^	−5.55^*∗∗*^	−4.32^*∗∗*^	−4.63^*∗*^	−22.82^*∗∗∗*^
*X*_2_^2^	−4.37^*∗*^	−3.56^*∗∗*^	−5.93^*∗∗*^	−0.80
*X*_3_^2^	−7.46^*∗∗*^	1.17	0.9999	−7.35^*∗∗*^

*F*-value (model)	31.33^*∗∗∗*^	61.66^*∗∗∗*^	87.93^*∗∗∗*^	76.60^*∗∗∗*^
*F*-value (lack of fit)	1.58	7.04	0.74	5.41
*R* ^2^	0.9758	0.9875	0.9912	0.9899
Adj-*R*^2^	0.9446	0.9715	0.9800	0.9770
Pred-*R*^2^	0.8086	0.8679	0.9372	0.9255
CV (%)	3.55	9.28	4.83	5.98

^*∗*^ *p* < 0.05, ^*∗∗*^*p* < 0.01, ^*∗∗∗*^*p* < 0.001.

**Table 4:** Experimental and predicted values of response variables at optimum extraction conditions.

Response variables	Optimum extraction conditions			Maximum value	
	*X*_1_ (%)	*X*_2_ (W)	*X*_3_ (min)	Experimental value	Predicted value
TPC (mg·GAE/g)	58	400	5	82.36 ± 1.05	83.90
TFC (mg·QE/g)				19.93 ± 2.50	21.84
DPPH (%)				73.61 ± 0.57	75.67
ABTS (%)				80.20 ± 3.23	82.66

*X*_1_: ethanol concentration (%); *X*_2_: microwave power (W); *X*_3_: extraction time (min). Experimental results were expressed as average values ± standard deviation (*n* = 3).

**Table 5:** Comparison of MAE with CSE.

Extraction method	Ethanol (%)	Time (min)	Power (W)	Temp (°C)	TPC (mg·GAE/g)	TFC (mg·QE/g)	DPPH (%)	ABTS (%)
MAE	58	5	400	–	82.36 ± 1.05^*a*^	19.93 ± 2.50^*a*^	73.61 ± 0.57^*a*^	80.20 ± 3.23^*a*^
CSE	56	23	–	63	51.86 ± 2.40^*b*^	11.14 ± 1.90^*b*^	60.56 ± 2.85^*b*^	63.82 ± 3.45^*b*^

The results are expressed as mean ± SD (*n* = 3). Values within the same column with different letters are significantly different at *p* < 0.05.

**Table 6:** HPLC quantification of phenolic compounds in avocado seed extract under optimal MAE conditions.

Compounds	Retention time (min)	Content (mg/100g·dw)
Gallic acid	3.32	6.89 ± 0.04
Catechin	9.65	52.46 ± 0.15
4-Hydroxybenzoic acid	10.45	12.47 ± 0.05
Vanillic acid	12.61	6.71 ± 0.01
Caffeic acid	12.90	4.18 ± 0.51
Syringic acid	13.36	45.87 ± 0.05
*p*-Coumaric acid	16.72	7.13 ± 0.22
Rutin	17.06	71.67 ± 2.04
Ferulic acid	17.92	4.76 ± 0.45
Quercetin	24.28	6.72 ± 0.02

## Data Availability

The data used to support the findings of this study are available from the corresponding author upon request.

## Supplementary Materials

Supplementary Materials Figure S1: gallic acid calibration curve. Figure S2: quercetin calibration curve.
