# Supplementary figures and images for: Simultaneous Optimization of Microwave-Assisted Extraction of Phenolic Compounds and Antioxidant Activity of Avocado (Persea americana Mill.) Seeds Using Response Surface Methodology
Source: J Anal Methods Chem. 2020 Aug 17;2020:7541927. doi: 10.1155/2020/7541927 (PMC7448120; doi:10.1155/2020/7541927)

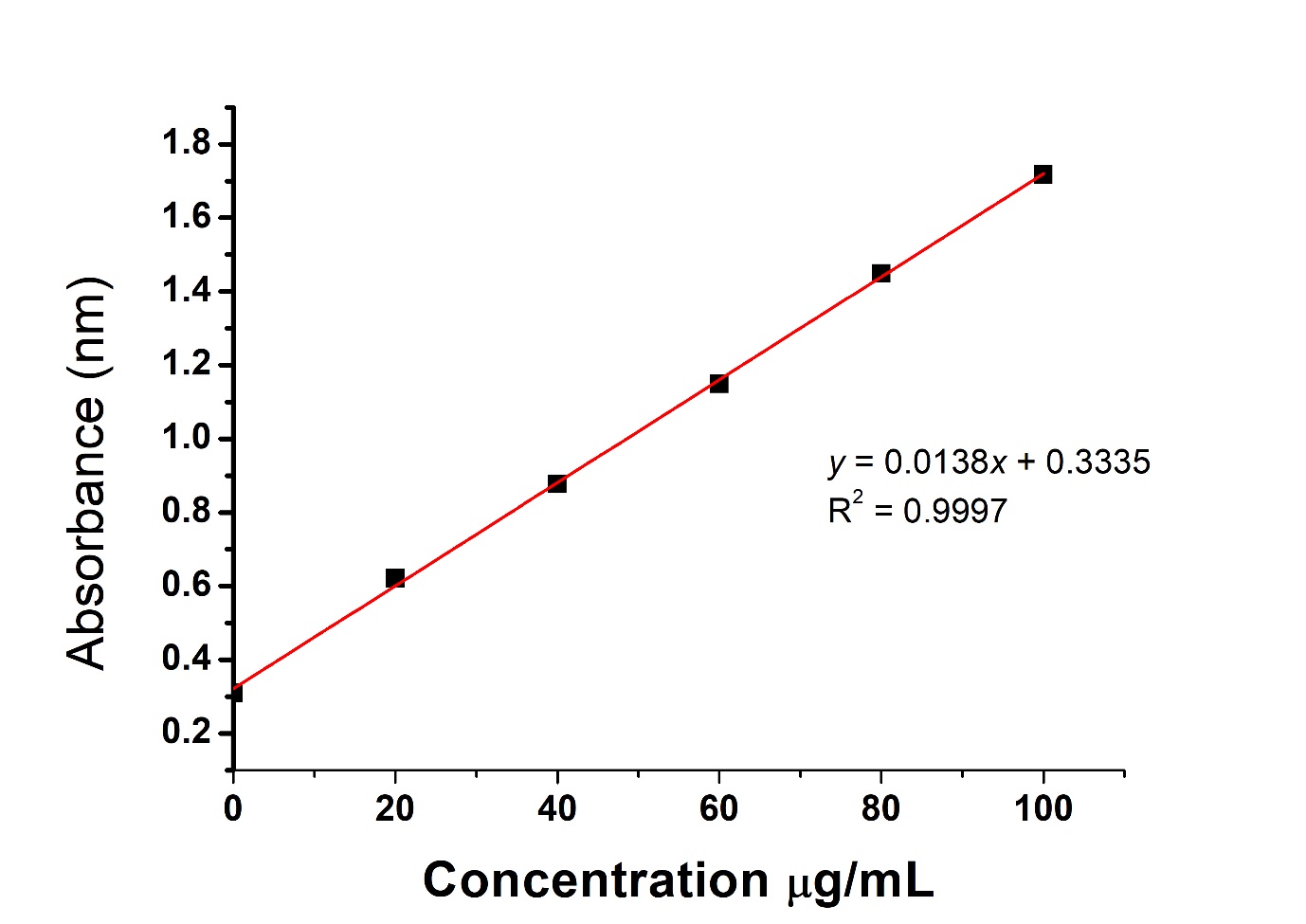


Figure S1: Gallic acid calibration curve


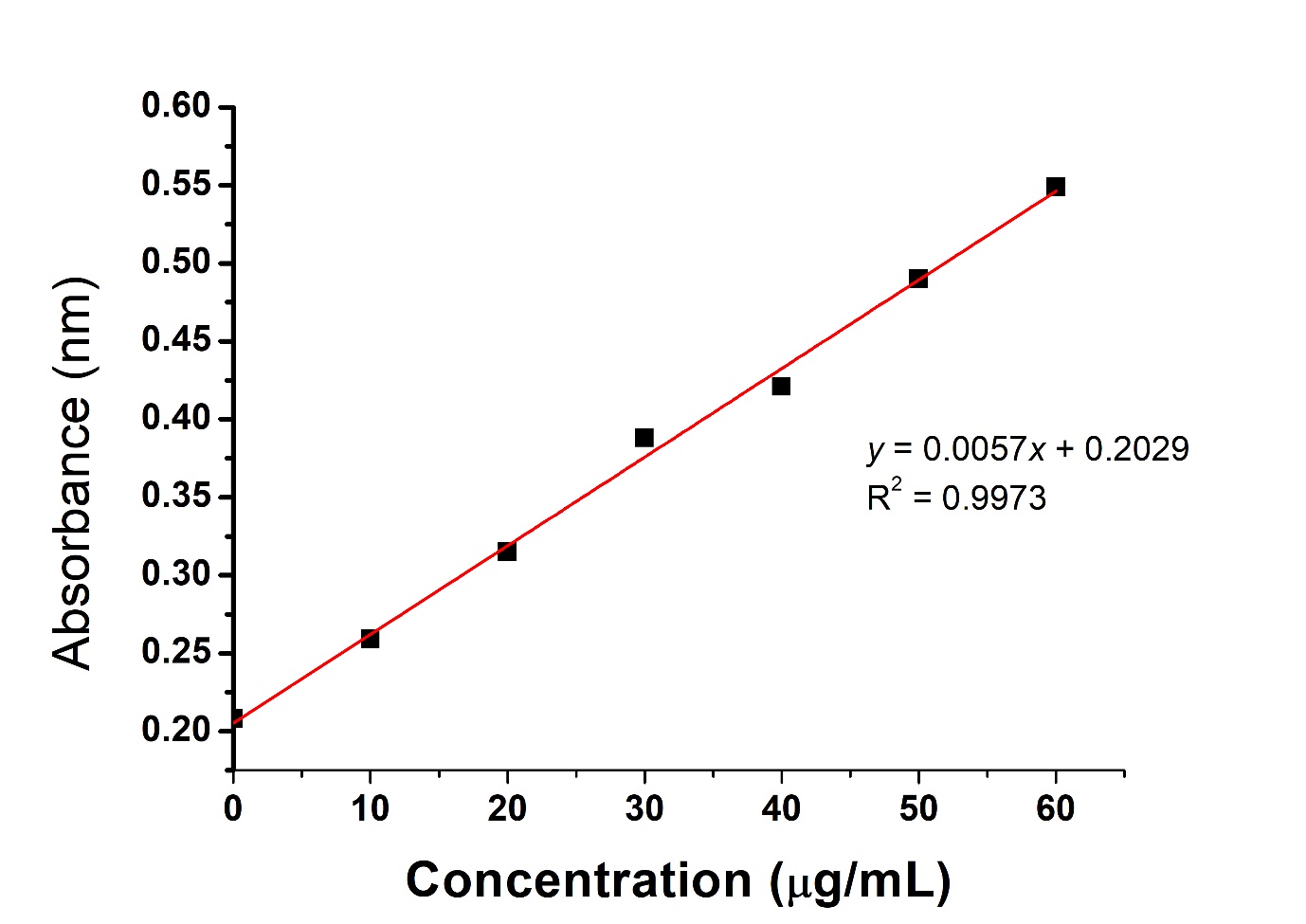


Figure S2: Quercetin calibration curve

Supplement: Supplementary Materials — Figure S1: gallic acid calibration curve. Figure S2: quercetin calibration curve. [file 7541927.f1.docx]
